# *Bifidobacterium longum* Depletion and Bile Acid Dysregulation in MASLD: Links to Hepatic TGR5–AMPK Signaling

**DOI:** 10.3390/microorganisms14102315

**Published:** 2026-10-10

**Authors:** Shaojun Li, Wenxiang Sheng, Yang Yang, Peixuan Li, Qing Su, Zhongtian Shi, Junyu Zhou, Heng Yuan, Sifang Feng

**Affiliations:** 1 Department of Respiratory and Critical Care Medicine, The First Affiliated Hospital of Xi’an Jiaotong University, Xi’an 710061, China; lishaojun0110@126.com; 2 Department of Physiology and Pathophysiology, School of Basic Medical Sciences, Xi’an Jiaotong University, Xi’an 710049, China; 2253211300@stu.xjtu.edu.cn (W.S.); 2254114656@stu.xjtu.edu.cn (Y.Y.); lpeix558811@outlook.com (P.L.); qingsuxjd2018@xjtu.edu.cn (Q.S.); medicinefiveszt@stu.xjtu.edu.cn (Z.S.); 3 Institute of Advanced Clinical Medicine, Peking University, Beijing 100191, China; benjaminjhou@bjmu.edu.cn

**Keywords:** metabolic dysfunction-associated steatotic liver disease (MASLD), gut microbiota, *Bifidobacterium longum*, secondary bile acids, TGR5, AMPK signaling, microbial co-occurrence network

## Abstract

Metabolic dysfunction-associated steatotic liver disease (MASLD) is the most common chronic liver disease worldwide. Although gut microbiota dysbiosis and aberrant bile acid metabolism are central to MASLD pathogenesis, the critical keystone species and precise molecular mechanisms remain elusive. Here, we performed an integrated analysis of 14 public 16S rRNA datasets (275 controls, 352 patients), combining multi-method differential abundance screening, machine learning, and network analysis to pinpoint MASLD-specific microbial signatures. We further validated the therapeutic potential of candidate secondary bile acids (SBAs) in a HepG2 steatosis model. MASLD patients displayed significantly reduced microbial diversity and a distinct community structure. *Bifidobacterium longum* emerged as the top discriminative putative keystone species, showing marked depletion and reduced network centrality. The secondary bile acid biosynthesis pathway was downregulated and positively correlated with B. longum abundance. Mechanistically, in vitro assays demonstrated that two SBAs, 23-nor-deoxycholic acid (23-NCA) and sodium taurodeoxycholate (TDCA-Na), alleviated hepatocellular lipid accumulation and inflammation, concomitant with TGR5 upregulation, AMPK activation, and p38 MAPK inhibition. In conclusion, depletion of the putative keystone species B. longum is associated with downregulated predicted genomic potential for secondary bile acid biosynthesis, while candidate SBAs protect hepatocytes in association with the activation of the hepatic TGR5–AMPK signaling axis in vitro. These findings offer a mechanistic basis for therapeutic strategies targeting the microbiota-bile acid axis.

## 1. Introduction

Metabolic dysfunction-associated steatotic liver disease (MASLD), the consensus nomenclature that superseded non-alcoholic fatty liver disease (NAFLD) [1], now represents the most prevalent chronic liver condition globally. Pooled prevalence estimates place MASLD at approximately 30% of the adult population, with incidence trajectories mirroring worldwide rises in obesity and metabolic syndrome [2,3]. The disease spectrum extends from simple steatosis through metabolic dysfunction-associated steatohepatitis (MASH) to advanced fibrosis, cirrhosis, and hepatocellular carcinoma, conferring heightened cardiovascular and hepatic mortality alongside an escalating socioeconomic burden [4]. Over the past decade, the gut microbiota has emerged as a pivotal environmental determinant of MASLD susceptibility and progression [5,6]. Metagenomic and 16S rRNA-based profiling studies have consistently demonstrated that patients with MASLD harbor a compositionally and functionally distinct gut microbial community relative to healthy controls, marked by diminished α-diversity, expansion of opportunistic pathobionts, and depletion of saccharolytic commensals [7,8]. Compromised intestinal barrier integrity further enables translocation of bacterial endotoxins and other microbe-associated molecular patterns into the portal circulation, thereby activating hepatic innate immune responses through the gut-liver axis [9,10].

Among the diverse metabolic transformations mediated by gut microbes, bile acid biotransformation ranks among the most physiologically consequential [11]. Primary bile acids, cholic acid (CA) and chenodeoxycholic acid (CDCA), are synthesized from cholesterol in hepatocytes, conjugated with glycine or taurine, and secreted into the intestinal lumen. There, microbial bile salt hydrolases (BSH) catalyze deconjugation, and subsequent 7α-dehydroxylation generates the principal secondary bile acids deoxycholic acid (DCA) and lithocholic acid (LCA) [12]. These secondary species function as potent signaling molecules through both nuclear receptors, such as the farnesoid X receptor (FXR), and membrane-bound receptors. Among the latter, the G protein-coupled bile acid receptor TGR5 (GPBAR1) has attracted particular interest as a metabolic and immunomodulatory hub [12,13]. TGR5 is robustly expressed in hepatocytes, brown adipose tissue, skeletal muscle, Kupffer cells, and intestinal enteroendocrine L-cells. Ligation of TGR5 by secondary bile acids activates cyclic adenosine monophosphate (cAMP)/protein kinase A (PKA) signaling cascades, promoting energy expenditure and improving glycaemic control [14,15]. In hepatocytes, TGR5 engagement has been mechanistically linked to AMP-activated protein kinase (AMPK) phosphorylation and activation [14,16]. AMPK is a central energy-sensing kinase that stimulates fatty acid β-oxidation via transcriptional upregulation of peroxisome proliferator-activated receptor gamma coactivator 1-α (PGC-1α) and peroxisome proliferator-activated receptor α/δ (PPARα/δ) [16,17]. In parallel, TGR5 signaling suppresses p38 mitogen-activated protein kinase (MAPK) phosphorylation, attenuating the release of pro-inflammatory cytokines including interleukin-1β (IL-1β) and tumor necrosis factor-α (TNF-α) [18,19].

Central to the generation of this bioactive secondary bile acid pool are specific bacterial consortia within the gut microbiota. *Bifidobacterium longum* is among the earliest and most abundant colonizers of the human gastrointestinal tract, contributing to carbohydrate fermentation, micronutrient biosynthesis, and competitive exclusion of pathogens [20]. Beyond these functions, *B. longum* expresses bile salt hydrolase (BSH) enzymes that deconjugate primary bile salts, a critical upstream step that liberates substrates for downstream microbial 7α-dehydroxylation [12,20]. Furthermore, *B. longum* participates in cross-feeding networks with butyrate-producing anaerobes, principally *Clostridium* species, by exporting acetate and lactate as carbon and energy sources that sustain their metabolic activity [21]. Specific *Clostridium* lineages within clusters XIVa and XIVb are the principal effectors of bile acid 7α-dehydroxylation, converting deconjugated primary bile acids into the bioactive secondary bile acids DCA and LCA [11,12]. The syntrophic partnership between *B. longum* and *Clostridium* species thus constitutes a critical metabolic axis governing the intestinal secondary bile acid supply available for host receptor engagement.

On the basis of these converging observations, we hypothesized that gut microbiota dysbiosis in MASLD patients results in concurrent depletion of *Bifidobacterium longum* and its *Clostridium* cross-feeding partners. This depletion would impair the predicted microbial capacity for secondary bile acid biosynthesis and potentially perturb the hepatic TGR5-AMPK signaling axis. The resulting mechanistic cascade would promote excessive hepatocyte lipid accumulation through reduced PGC-1α/PPARα/δ-mediated fatty acid β-oxidation and sustain chronic hepatic inflammation via unchecked p38 MAPK signaling. To test this hypothesis, we performed a systematic bioinformatic reanalysis of publicly deposited 16S rRNA gene amplicon sequencing datasets from healthy individuals and MASLD patients. Using an integrated analytical framework comprising microbial diversity profiling, differential abundance testing, machine learning-based biomarker discovery, co-occurrence network analysis, and functional pathway inference, we sought to define the microbial taxa and metabolic functions that mechanistically link gut dysbiosis to impaired secondary bile acid metabolism in MASLD.

## 2. Methods

### 2.1. Bioinformatic Reanalysis of Gut Microbiota Data

#### 2.1.1. Study Design

We performed a PRISMA-guided systematic reanalysis of publicly available 16S rRNA amplicon datasets to compare gut microbiome composition between healthy individuals and patients with metabolic dysfunction-associated steatotic liver disease (MASLD) [22]. Studies were identified through searches of the European Molecular Biology Laboratory European Bioinformatics Institute (EMBL-EBI), the National Center for Biotechnology Information (NCBI), and the Gut Microbiota Repository (GMrepo), encompassing publications through February 2026. Search terms included ‘non-alcoholic fatty liver disease’ (NAFLD), ‘metabolic dysfunction-associated steatotic liver disease’ (MASLD), ‘secondary bile acids’, ‘gut microbiota’, and their Medical Subject Headings (MeSH) equivalents.

Studies were included when MASLD diagnosis adhered to the multisociety Delphi consensus statement on fatty liver disease nomenclature [1] and when institutional review board approval with informed consent had been obtained. To minimize confounding, we excluded studies in which participants were younger than 18 years, had comorbid viral hepatitis, hypertension, diabetes mellitus, or cardiovascular disease, or had received antibiotics within the preceding three months. Only studies using 16S rRNA gene amplicon sequencing as the primary profiling method were retained.

#### 2.1.2. Data Processing

Raw 16S rRNA sequencing data were retrieved from the Sequence Read Archive (SRA) and converted to paired-end FASTQ format using the SRA Toolkit (v3.1.1). All datasets were processed through a unified pipeline in QIIME 2 (version 2025.2) [23]. Quality control removed sequences with ambiguous bases and enforced a minimum Phred quality score of 30, with a per-sample read depth threshold of 10,000 reads. To resolve regional heterogeneity across studies, cohorts originally spanning the V3–V4 region were computationally extracted and trimmed in silico to the homologous V4 hypervariable region, standardizing the target locus across all datasets prior to downstream inference. Amplicon sequence variants (ASVs) were generated using the DADA2 plugin (v1.40) [24] with denoising, consensus-based chimera removal, and truncation at 240 bp (forward) and 200 bp (reverse). Taxonomic assignment used a pre-trained naive Bayes classifier against the SILVA 138.2 database [25], targeting the V4 hypervariable region at 99% sequence similarity. Only ASVs present at a minimum frequency of 10 across all samples were retained for downstream analysis.

To address inter-cohort heterogeneity, all datasets were processed through the same pipeline to minimize technical variability. We did not apply formal batch correction tools such as ComBat or MMUPHin; instead, two complementary strategies were used. First, uniform quality-control filtering, in silico target-region harmonization to the V4 region, and identical DADA2 denoising parameters were enforced across all raw data to eliminate upstream technical divergence. Second, downstream biomarker discovery was restricted to taxa mutually validated across four orthogonal statistical and machine-learning frameworks (LEfSe, version 1.1.2; STAMP, version 2.1.3; ALDEx2, version 1.40.0; and XGBoost, version 3.0.0), thereby minimizing reliance on single-cohort technical artifacts or model-specific distributional assumptions.

### 2.2. Microbiome Profiling and Multi-Omics Bioinformatic Analysis

#### 2.2.1. Microbiome Profiling

Bioinformatic and statistical analyses were performed in R (v4.2.1) [26]. Within-sample diversity (α-diversity) was quantified using the Chao1 index (species richness), the Shannon diversity index (reflecting richness and evenness), and the Simpson index (community evenness and dominance). To evaluate between-group differences in overall microbial community composition, partial least squares discriminant analysis (PLS-DA) was conducted using the FactoMineR (v2.11) [27] and metaX (v2.2.1) [28] packages. Model performance and predictive ability were evaluated by goodness-of-fit metrics alongside cross-validation (reporting R2Y and Q2 values), and the statistical significance of separation between cohorts was determined using a permutation test (*p* < 0.001, 200 iterations). All graphical representations and ordination plots were generated using the ggplot2 package (v3.5.1) [29].

#### 2.2.2. Biomarker Discovery

To identify microbial features associated with MASLD, we applied a multi-layered filtering and machine learning framework. Amplicon sequence variants (ASVs) with relative abundance below 0.1% were first excluded to reduce background noise. Differential abundance across disease stages was screened with linear discriminant analysis effect size (LEfSe) [30], which combines the Kruskal–Wallis test with subsequent linear discriminant analysis.

An extreme gradient boosting (XGBoost) classifier [31] was then trained on the filtered microbial features to predict disease status. Model interpretability was enhanced with SHapley Additive exPlanations (SHAP) [32], which computes mean absolute SHAP values for each ASV at the species level, revealing relative contributions to classification. Five-fold cross-validation was applied during training to assess performance stability and reduce overfitting. Independent cohort validation was not performed owing to the absence of external datasets with compatible metadata and sequencing protocols.

#### 2.2.3. Network Construction

Microbial co-occurrence networks were constructed using FastSpar (v1.0.3) [33], which estimates Sparse Correlations for Compositional data (SparCC) between microbial taxa. The resulting network was visualized in Gephi (v0.9.5) [34].

#### 2.2.4. Functional Prediction

Functional potential of the gut microbiome was predicted with PICRUSt2 (v2.5.3) [35], which maps 16S rRNA gene sequences to reference genomes in the KEGG database [36]. Functional profiles were Z-score normalized. To explore relationships between microbial biomarkers and biological functions, correlation analyses were performed using ggplot2 [29] and the linkET package (v0.0.3), supplemented by Mantel tests to assess inter-matrix correlations. The pathogenic impact of specific microorganisms was further quantified using the DCiPatho method [37], which computes the product of relative abundance and virulence scores across conditions.

### 2.3. In Vitro Validation Using a HepG2 Hepatic Steatosis Model

#### 2.3.1. Establishment of an In Vitro Hepatic Steatosis Model

To experimentally validate the pathogenic relevance of the bile acid metabolites identified through the above bioinformatic analyses, an in vitro hepatocyte injury model was further established using the human hepatocellular carcinoma cell line HepG2. Cells were routinely cultured and subcultured in high-glucose DMEM supplemented with 10% fetal bovine serum, 1% L-glutamine, and 100 U/mL penicillin-streptomycin, and maintained at 37 °C in a humidified atmosphere containing 5% CO_2_. Cells in the logarithmic growth phase were seeded into 6-well plates at a density of 3.5 × 10^5^ cells per well [38]. Upon reaching 70–80% confluence, cells were co-treated with oleic acid (OA) and lipopolysaccharide (LPS) for 24 h to induce hepatocellular steatosis accompanied by inflammatory injury [39]. After model induction, the medium was replaced with fresh medium, and cells were treated for 24 h with five representative candidate bile acid species (each at 10 μM, vehicle: DMSO < 0.1% *v*/*v*, n = 5 independent biological replicates), selected based on the literature-reported differential abundance and metabolic relevance in non-alcoholic steatohepatitis/MASLD cohorts: 7-ketodeoxycholic acid (7-KDCA), sodium taurodeoxycholate (TDCA-Na), chenodeoxycholic acid-3-glucuronide (CDCA-3G), 23-nor-deoxycholic acid (23-NCA), and ursodeoxycholic acid-derived conjugate/species (UNCA), alongside untreated blank control and OA+LPS-treated model groups.

#### 2.3.2. Assessment of Lipid Deposition, Inflammatory Response, and Proteomic Profiling

At the end of the intervention, cellular and supernatant samples were collected for the following analyses. Oil Red O and hematoxylin–eosin (HE) staining were performed to qualitatively assess intracellular lipid droplet accumulation and the extent of cellular morphological injury, respectively [40]. Intracellular triglyceride (TG) content was determined using a TG assay kit ( Asan Pharmaceutical, Asan, Republic of Korea). The secretion levels of inflammatory cytokines in the culture supernatant were measured by enzyme-linked immunosorbent assay (ELISA) using commercial kits (Crystal Chem, Itasca, IL, USA). mRNA and protein expression levels of target genes were assessed by quantitative real-time PCR (qRT-PCR) and Western blotting, respectively. For Western blotting, four independent biological replicates were prepared per group; owing to the limited protein yield from individual samples, equal amounts of protein from two biological replicates were pooled pairwise before electrophoresis, yielding two pooled samples per group for Western blotting and densitometric analysis. In parallel, cell samples (n = 3) were subjected to data-independent acquisition (DIA) quantitative proteomics. Cells were lysed in 8 M urea buffer (100 mM TEAB, pH 8.5), reduced with DTT, alkylated with iodoacetamide, and digested with trypsin (allowing ≤2 missed cleavages), followed by C18 desalting. Peptides mixed with iRT standards were separated on an Evosep One UHPLC system coupled to a Q Exactive HF-X mass spectrometer (Thermo Fisher Scientific, Bremen, Germany). DDA library generation was conducted in Proteome Discoverer 2.2 against the customized database (1131721-1.fasta, 27,275 sequences; precursor tolerance: 10 ppm, fragment tolerance: 0.02 Da), and DIA quantification was performed using Spectronaut(version 19; Biognosys AG, Schlieren, Switzerland). Strict thresholds were applied: FDR < 1% at both peptide and protein levels (≥1 unique peptide, Q-value < 0.01). Protein abundance was normalized by total peptide peak area. Differentially expressed proteins were filtered with fold change ≥2.0 or ≤0.5 and Student’s *t*-test *p* ≤ 0.05. GO and KEGG functional enrichments were evaluated by hypergeometric test (*x*/*n* > *y*/*N*, *p* < 0.05). The datasets generated during the proteomic profiling are available from the corresponding author upon reasonable request. [41].

### 2.4. Statistical Analysis

All statistical analyses were performed in SPSS (version 27.0; SPSS Inc., Armonk, NY, USA) [42]. Unless otherwise specified, data from in vitro experiments are presented as mean ± standard deviation (SD) of five independent biological replicates (n = 5). Between-group differences were assessed by one-way analysis of variance (ANOVA) followed by Bonferroni-corrected post hoc comparisons. Statistical significance was set at *p* < 0.05.

## 3. Results

### 3.1. Study Selection and Clinical Characteristics of MASLD Cohorts

**Figure 1:**
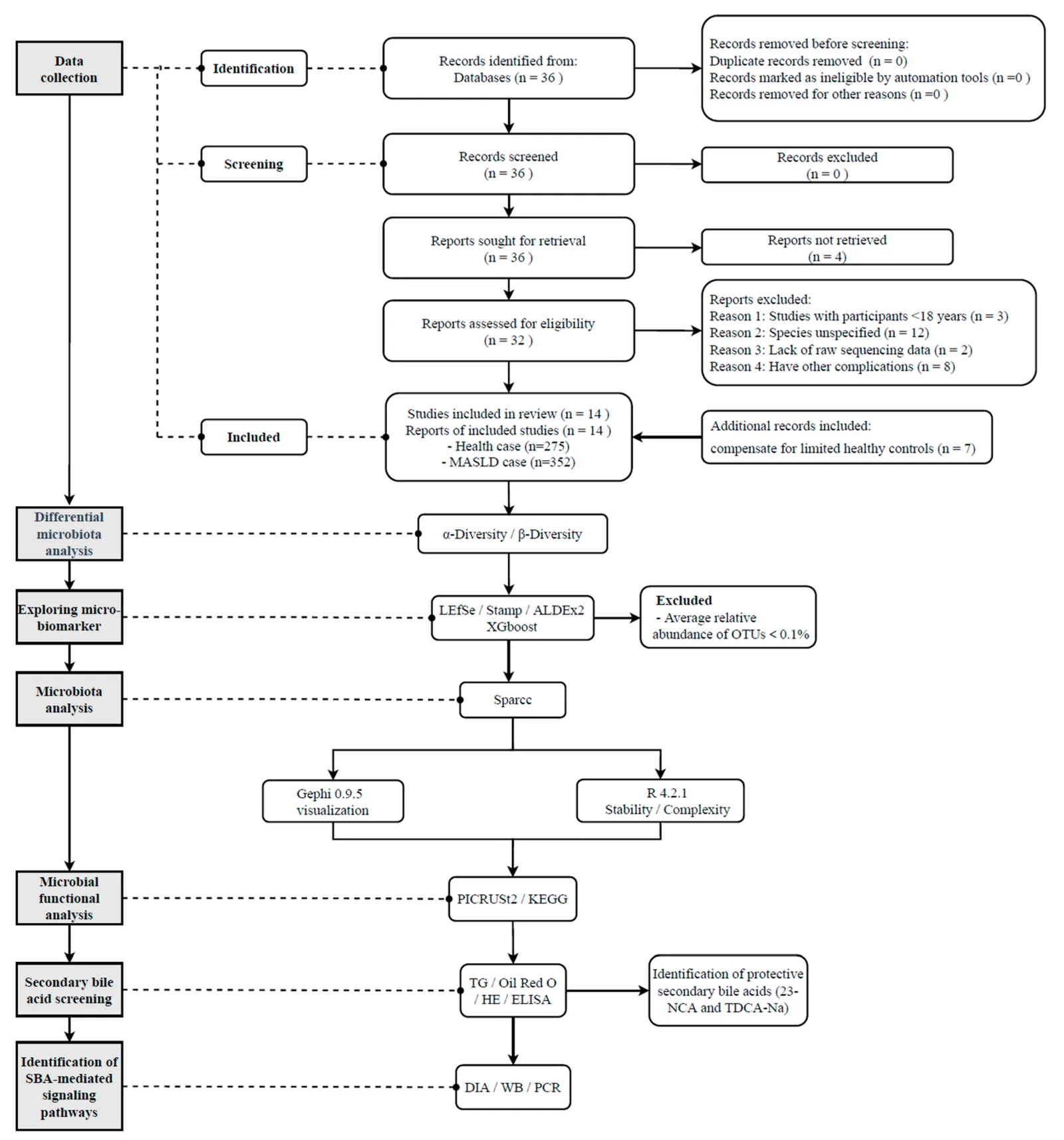
Study design and analytical workflow for gut microbiota reanalysis and experimental validation in MASLD. Schematic overview of the study workflow. Data collection: publicly available 16S rRNA gene amplicon datasets were identified and screened following PRISMA guidelines. Of 36 potentially relevant studies retrieved for detailed evaluation, 29 were excluded (reports not retrieved, n = 4, participants aged <18 years, n = 3; unspecified species-level taxonomic annotation, n = 12; lack of raw sequencing data, n = 2; presence of unrelated comorbidities, n = 8). To compensate for the limited sample size of healthy controls, 7 additional cohorts comprising 167 healthy volunteers were included, yielding 14 eligible studies comprising 275 healthy controls and 352 patients with MASLD for downstream analysis. Differential microbiota analysis: gut microbial α- and β-diversity were compared between groups. Exploring microbial biomarkers: differentially abundant taxa discriminating MASLD from healthy controls were identified using LEfSe, STAMP, ALDEx2, and XGBoost, after excluding amplicon sequence variants (ASVs) with average relative abundance <0.1%. Microbiota analysis: microbial co-occurrence networks were constructed using SparCC, visualized with Gephi (v0.9.5), and evaluated for network stability and complexity in R (v4.2.1). Microbial functional analysis: the functional potential of the gut microbiota was predicted using PICRUSt2 and annotated against the KEGG database. Secondary bile acid (SBA) screening: candidate SBAs were evaluated for hepatoprotective activity in an oleic acid (OA)/lipopolysaccharide (LPS)-induced HepG2 steatosis model using triglyceride (TG) quantification, Oil Red O staining, hematoxylin–eosin (HE) staining, and enzyme-linked immunosorbent assay (ELISA), identifying 23-NCA and TDCA-Na as protective SBAs. Identification of SBA-mediated signaling pathways: the downstream signaling pathways mediating the hepatoprotective effects of the identified SBAs were characterized using data-independent acquisition (DIA) quantitative proteomics, Western blotting (WB), and quantitative real-time PCR (qRT-PCR).

The study selection workflow is illustrated in Figure 1. All included studies conformed to the updated MASLD nomenclature and met the diagnostic criteria established by the American Association for the Study of Liver Diseases and the European Association for the Study of the Liver, with MASLD confirmed by imaging or biopsy. Initial screening identified 36 studies, of which 22 were excluded for the following reasons: one involved comorbidities unrelated to MASLD, three enrolled participants under 18 years of age, four lacked gut microbiome data, two lacked raw sequencing data, and twelve had incomplete species-level taxonomic annotation. The final analysis included 7 studies (accession numbers: PRJNA541489, PRJNA246121, PRJNA518731, PRJNA382861, PRJNA559052, PRJNA860335, and PRJEB40538), comprising 108 healthy volunteers and 352 patients with MASLD. Seven additional cohorts (PRJNA445763, PRJNA431746, PRJNA449353, PRJNA471972, PRJEB41867, PRJNA736583, and PRJNA748675), contributing 167 healthy volunteers, were included to compensate for the limited sample size of healthy controls. In total, 14 eligible studies were included, including 275 healthy controls and 352 MASLD patients, for follow-up analysis. The main characteristics of the included studies are summarized in Table 1. These studies have been subject to an ethical review by the Institute’s Ethics Committee and are in compliance with the Declaration of Helsinki (Supplementary File). Subgroup analysis and enterotype-based clustering were applied to reduce inter-cohort variability and to evaluate microbial community structure independently of technical batch effects.

### 3.2. Gut Microbiota Diversity and Differential Species in MASLD

**Figure 2:**
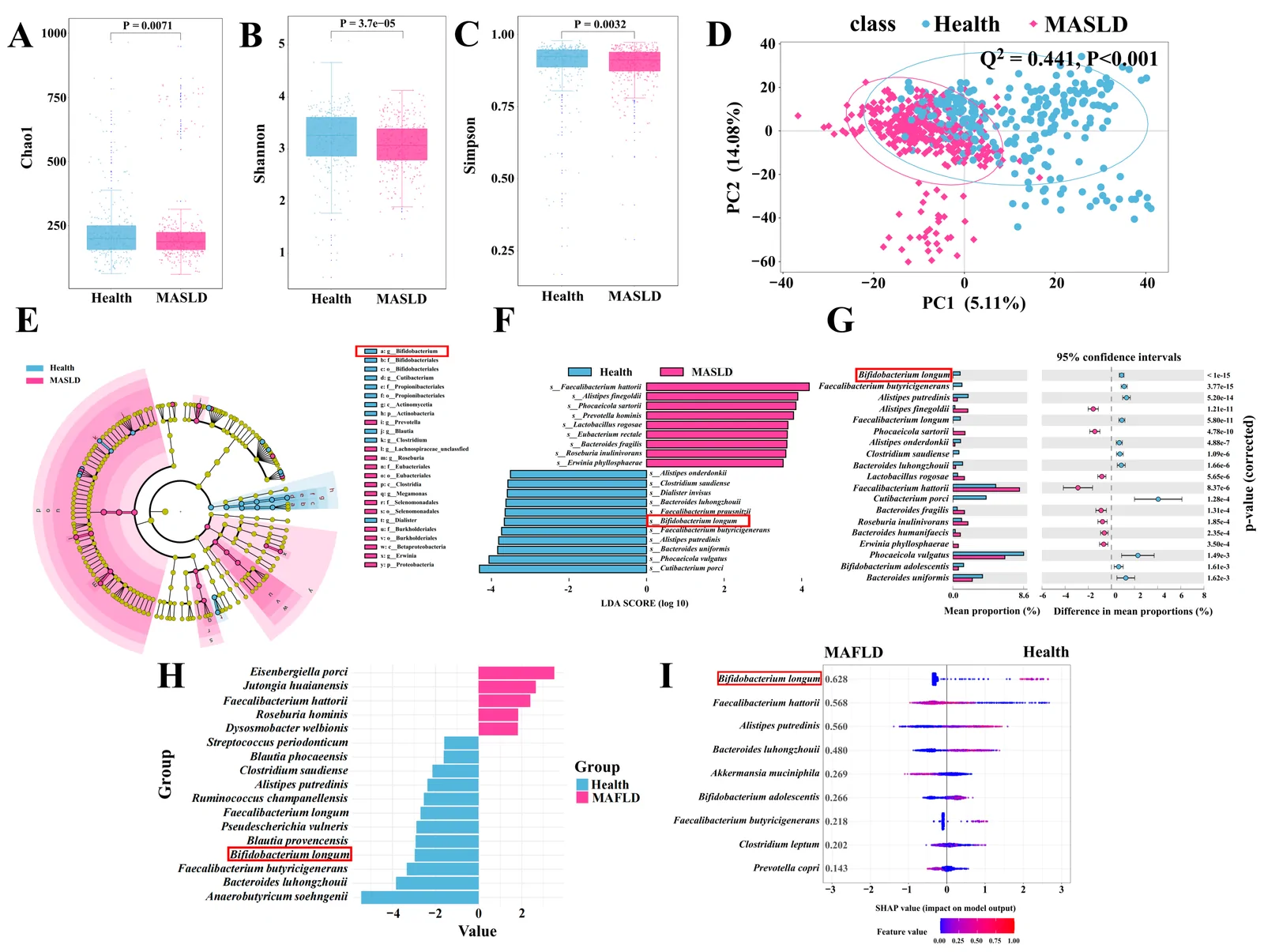
Gut microbiota characteristics in patients with MASLD compared with healthy controls. (**A**-**C**) α-diversity indices of the gut microbiota. (**A**) Chao1 index (species richness), (**B**) Shannon index (species diversity), and (**C**) Simpson index (species evenness). Blue, healthy controls; pink, MASLD. Statistical comparisons were performed using the Wilcoxon rank-sum test. (**D**) Partial least squares discriminant analysis (PLS-DA) based on Bray–Curtis distances showing separation of gut microbial community structure between the two groups. (**E**,**F**) Identification of differentially abundant taxa by linear discriminant analysis effect size (LEfSe), (**E**) cladogram of differentially abundant taxa and (**F**) histogram of linear discriminant analysis (LDA) scores (LDA threshold > 2.0). (**G**) STAMP analysis showing the relative abundance differences and 95% confidence intervals of key differential taxa. (**H**) ALDEx2 differential abundance analysis validating taxa significantly altered between the two groups. (**I**) Ranking of the most discriminative taxa by SHapley Additive exPlanations (SHAP) values from the XGBoost machine learning model; *Bifidobacterium longum* is highlighted with a red box.

To characterize the gut microbiota alterations associated with MASLD, we reanalyzed 16S rRNA gene V4 region sequencing and conducted a systematic comparative analysis between patients with MASLD and healthy controls (Figure 2). α diversity analysis revealed that patients with MASLD exhibited significantly reduced species richness (Chao1 index, *p* = 0.0071; Figure 2A), species diversity (Shannon index, *p* = 3.7 × 10^−5^; Figure 2B), and species evenness (Simpson index, *p* = 0.0032; Figure 2C) compared with healthy controls. Partial Least Squares Discriminant Analysis (PLS-DA) based on Bray–Curtis distances confirmed a marked separation of gut microbial community structure between the two groups, with robust model predictive performance (Q^2^ = 0.441, permutation test *p* < 0.001; Figure 2D).

To identify core differential species with high confidence, we applied four complementary analytical methods, LEfSe, STAMP, ALDEx2, and XGBoost machine learning, for cross-validation. LEfSe analysis (LDA threshold > 2.0; Figure 2E,F) and STAMP analysis (Figure 2G) jointly identified 19 differentially abundant species. Healthy controls were enriched in beneficial bacteria, most notably *Bifidobacterium longum*, whereas the MASLD group showed enrichment of multiple potentially pathogenic species. ALDEx2 analysis independently validated these differential abundance patterns (Figure 2H). XGBoost-based feature importance ranking using SHAP values identified *Bifidobacterium longum* as the most discriminative species between MASLD and healthy controls (SHAP = 0.628), followed by *Faecalibacterium hattorii* (SHAP = 0.568) and *Alistipes putredinis* (SHAP = 0.560) (Figure 2I).

**Table 1:** The characteristics of each selected case–control study for Metabolic Dysfunction-−Associated Steatotic Liver Disease (MASLD).

Study (ID)	Year	Instrument	Total	Health	MASLD	Age	Gender (M/F)	City, Country
PRJNA541489	2019	Illumina MiSeq	46	22	24	64.04 ± 7.30	33/13	Beijing, China
PRJNA246121	2015	Illumina HiSeq 2000	85	32	53	45.00 ± 10.17	32/53	Beijing, China
PRJNA518731	2020	Illumina NovaSeq	35	0	35	–	–	Auckland, New Zealand
PRJNA382861	2017	Illumina MiSeq	56	25	31	37.00 ± 6.33	22/34	Shanghai, China
PRJNA559052	2020	Illumina HiSeq	86	0	86	50.80 ± 12.60	56/30	Leuven, Belgium
PRJNA860335	2022	–	61	29	32	–	–	Beijing, China
PRJEB40538	2021	Illumina MiSeq	91	0	91	48.8 ± 9.36	53/38	Italy; Greece; Serbia
PRJNA445763	2018	–	20	20	0	–	–	Harbin, China
PRJNA431746	2018	Illumina HiSeq	8	8	0	–	–	Styria, Austria
PRJNA449353	2019	–	5	5	0	–	–	Nanjing, China
PRJNA471972	2020	Illumina HiSeq 4000	14	14	0	64.74 ± 9.80	–	Rome, Italy
PRJEB41867	2020	Illumina HiSeq	98	98	0	24.1 ± 12.65	48/50	Graz, Austria
PRJNA736583	2022	Illumina HiSeq	21	21	0	53.2 ± 8.34	–	Naples, Italy
PRJNA748675	2022	–	1	1	0	–	–	Taoyuan, China
Total			627	275	352			

MASLD, Metabolic dysfunction-associated steatotic liver disease; M: male; F: female.

### 3.3. Co-Occurrence Network and Functional Metabolic Pathway Alterations

**Figure 3:**
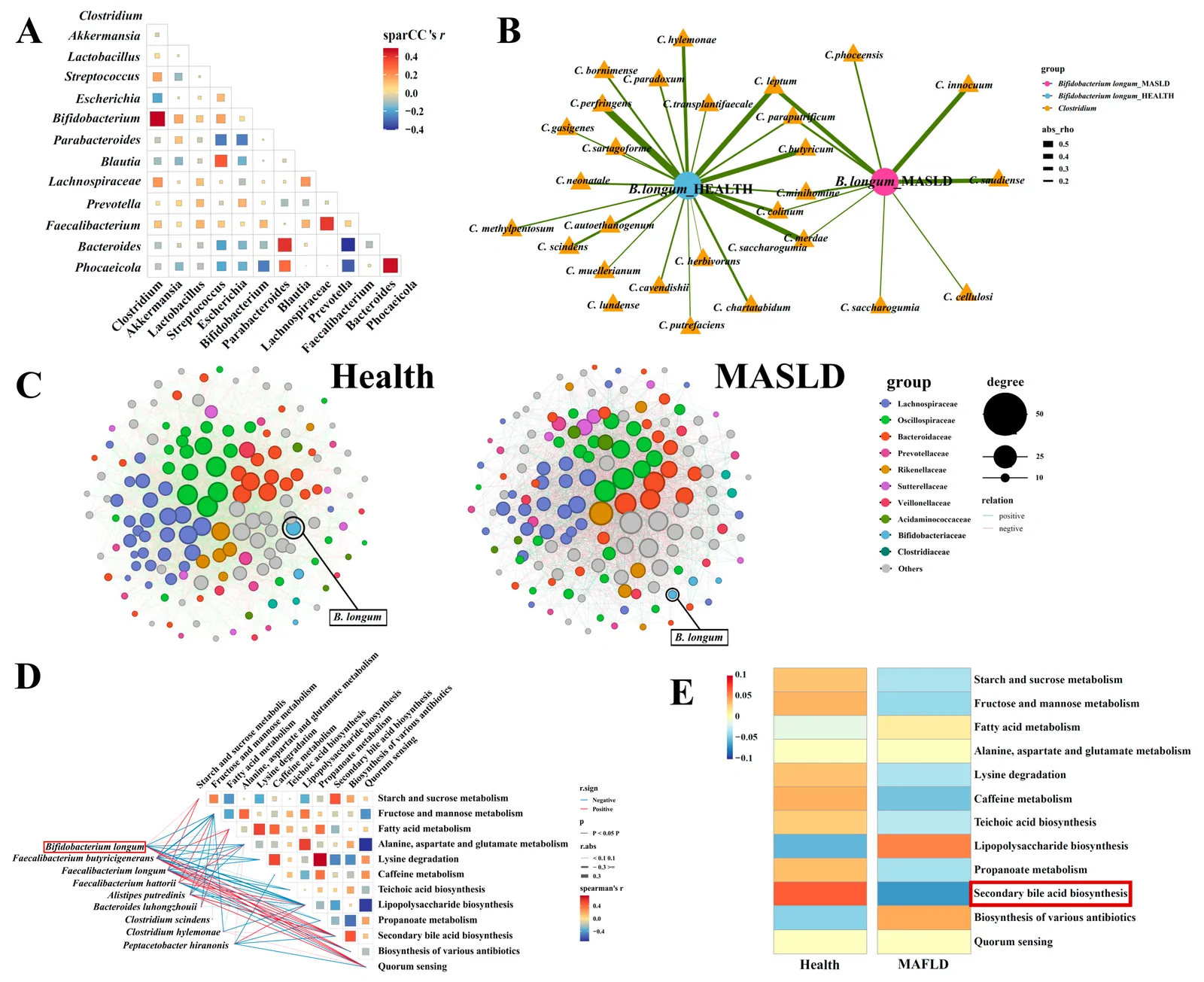
Gut microbial co-occurrence network, species-level correlations, and functional analysis. (**A**) Heatmap of Spearman correlations among dominant bacterial genera; the color gradient indicates the correlation coefficient (r value). (**B**) Core species-level co-occurrence subnetwork showing co-occurrence relationships among species; node size represents relative species abundance, and only positive correlations (green edges) are retained. (**C**) Overall gut microbial co-occurrence networks in healthy controls and patients with MASLD; nodes are colored by phylum, and node size is proportional to relative species abundance. (**D**) Correlation heatmap between core differential taxa and predicted KEGG functional pathways; *Bifidobacterium longum* is highlighted with a red box. (**E**) Heatmap of differentially enriched functional pathways predicted by PICRUSt2; the color gradient indicates relative pathway abundance, with the secondary bile acid (SBA) biosynthesis pathway highlighted by a red box.

To further investigate the inter-species interaction patterns and functional metabolic shifts in the MASLD gut microbiota, we constructed co-occurrence networks and performed functional prediction analysis. Spearman correlation analysis revealed the strongest positive correlation between *Bifidobacterium* and *Clostridium* (Figure 3A), and the core species co-occurrence subnetwork reflected this strong positive co-occurrence association (Figure 3B). Global co-occurrence network analysis (Figure 3C and Table 2) demonstrated that, compared with healthy controls, the MASLD gut microbiota network exhibited an increased average shortest path length (1.825 vs. 1.782), reduced network density (0.223 vs. 0.242), and elevated network heterogeneity (0.630 vs. 0.587) and centralization (0.436 vs. 0.370), collectively indicating weakened inter-bacterial interactions and diminished network stability. Notably, *Bifidobacterium longum*, a central topological hub in the healthy gut microbiota network, showed a marked decline in degree centrality (from 34 to 20) and betweenness centrality (from 0.023 to 0.009), and its proportion of negative interactions increased from 46.25% to 53.10%, indicating a marked shift in its topological prominence within the MASLD network.

Correlation analysis between core differential species and predicted KEGG functional pathways revealed that *Bifidobacterium longum* abundance was significantly positively correlated with the secondary bile acid (SBA) biosynthesis pathway (Figure 3D). PICRUSt2 functional prediction further confirmed that the SBA biosynthesis pathway was significantly downregulated in the MASLD gut microbiota, accompanied by upregulation of starch and sucrose metabolism, fructose and mannose metabolism, and pro-inflammatory pathways including lipopolysaccharide biosynthesis and quorum sensing (Figure 3E).

### 3.4. In Vitro Validation of Hepatoprotective Secondary Bile Acids

#### 3.4.1. Screening of Secondary Bile Acids for Hepatoprotective Effects

**Figure 4:**
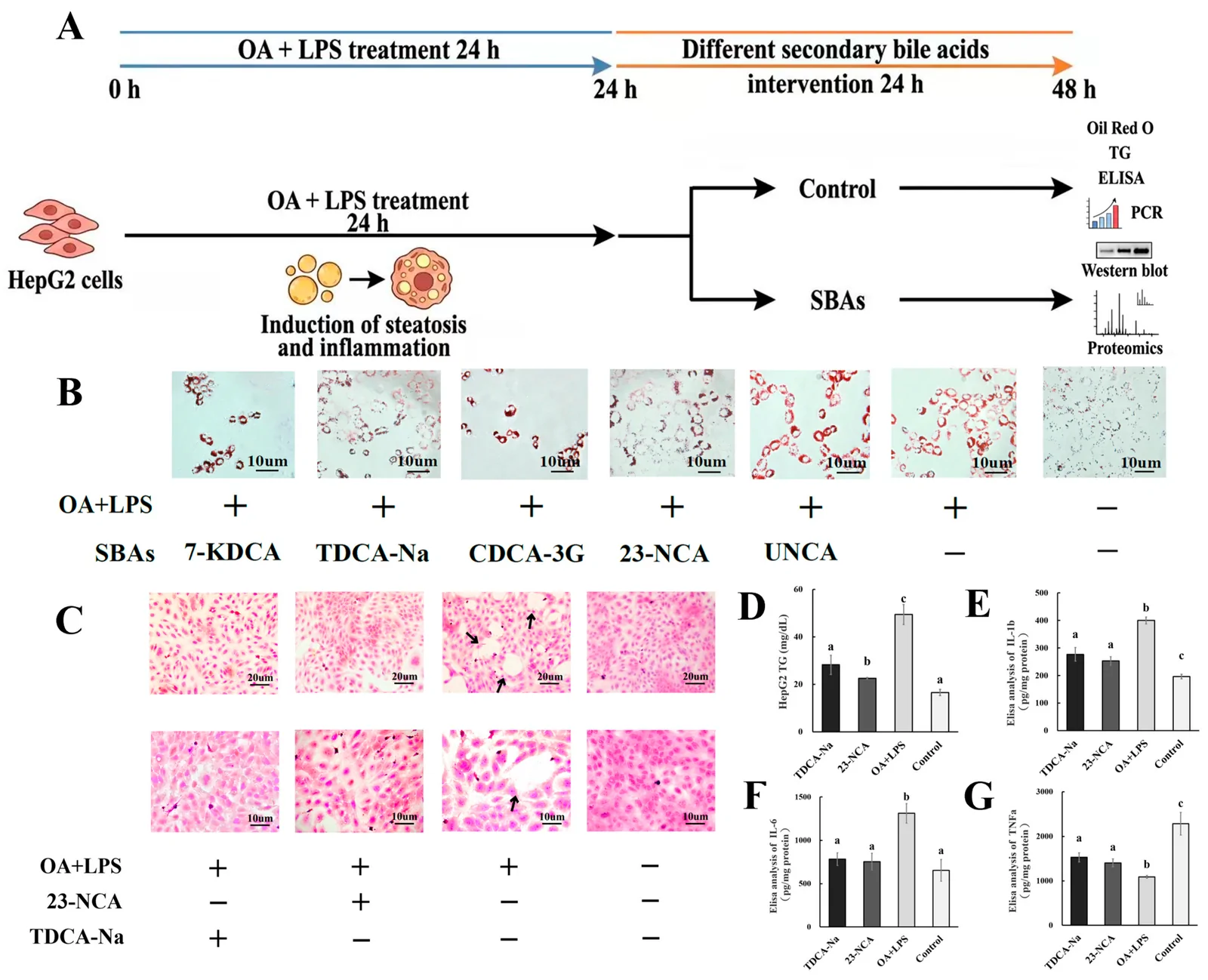
Protective effects of secondary bile acids (SBAs) against hepatocyte injury. (**A**) Schematic of the experimental design. HepG2 cells were treated with oleic acid (OA) plus lipopolysaccharide (LPS) for 24 h to induce steatosis and inflammation, followed by treatment with individual secondary bile acids for an additional 24 h. (**B**) Oil Red O staining showing intracellular lipid droplet accumulation; scale bar = 10 μm. (**C**) Hematoxylin–eosin (HE) staining showing hepatocellular morphological changes; black arrows indicate representative features of vacuolar degeneration (intracellular vacuolation) and cellular swelling; scale bars = 20 μm (upper row, low magnification) and 10 μm (lower row, high magnification zoom-in). (**D**) Intracellular triglyceride (TG) content in HepG2 cells. (**E**–**G**) Secretion levels of pro-inflammatory cytokines in the cell culture supernatant measured by enzyme-linked immunosorbent assay (ELISA): (**E**) IL-1β, (**F**) IL-6, and (**G**) TNF-α. For (**D**–**G**), different letters indicated significant differences between groups (*p* < 0.05), (n = 5).

Based on the clinical finding that the SBA biosynthesis pathway was significantly suppressed in MASLD, we established an in vitro MASLD hepatocyte injury model using HepG2 cells treated with oleic acid (OA) combined with lipopolysaccharide (LPS) for 24 h to induce steatosis and inflammation, followed by treatment with individual secondary bile acids for an additional 24 h (Figure 4A). Oil Red O staining showed that 7-KDCA, CDCA-3G, and UNCA had no appreciable effect on intracellular lipid droplet accumulation, whereas 23-NCA and TDCA-Na qualitatively attenuated lipid droplet formation (Figure 4B), which was quantitatively corroborated by the significant reduction in intracellular triglyceride (TG) levels (*p*< 0.05; Figure 4D). Hematoxylin and eosin (H&E) staining confirmed that OA+LPS treatment induced pronounced hepatocellular swelling, vacuolar degeneration, and blurred cell boundaries; both 23-NCA and TDCA-Na treatment partially alleviated these pathological changes and improved hepatocyte morphology (Figure 4C). Enzyme-linked immunosorbent assay (ELISA) results demonstrated that 23-NCA and TDCA-Na significantly reduced the secretion of pro-inflammatory cytokines IL-1β, IL-6, and TNF-α in the cell culture supernatant compared with the OA+LPS model group (Figure 4E–G). These results suggest that 23-NCA and TDCA-Na exert moderate protective effects in vitro, mitigating pro-inflammatory cytokine secretion while partially attenuating intracellular lipid accumulation in hepatocytes.

#### 3.4.2. Hepatoprotective Secondary Bile Acids Are Associated with TGR5 Upregulation and AMPK Activation

**Figure 5:**
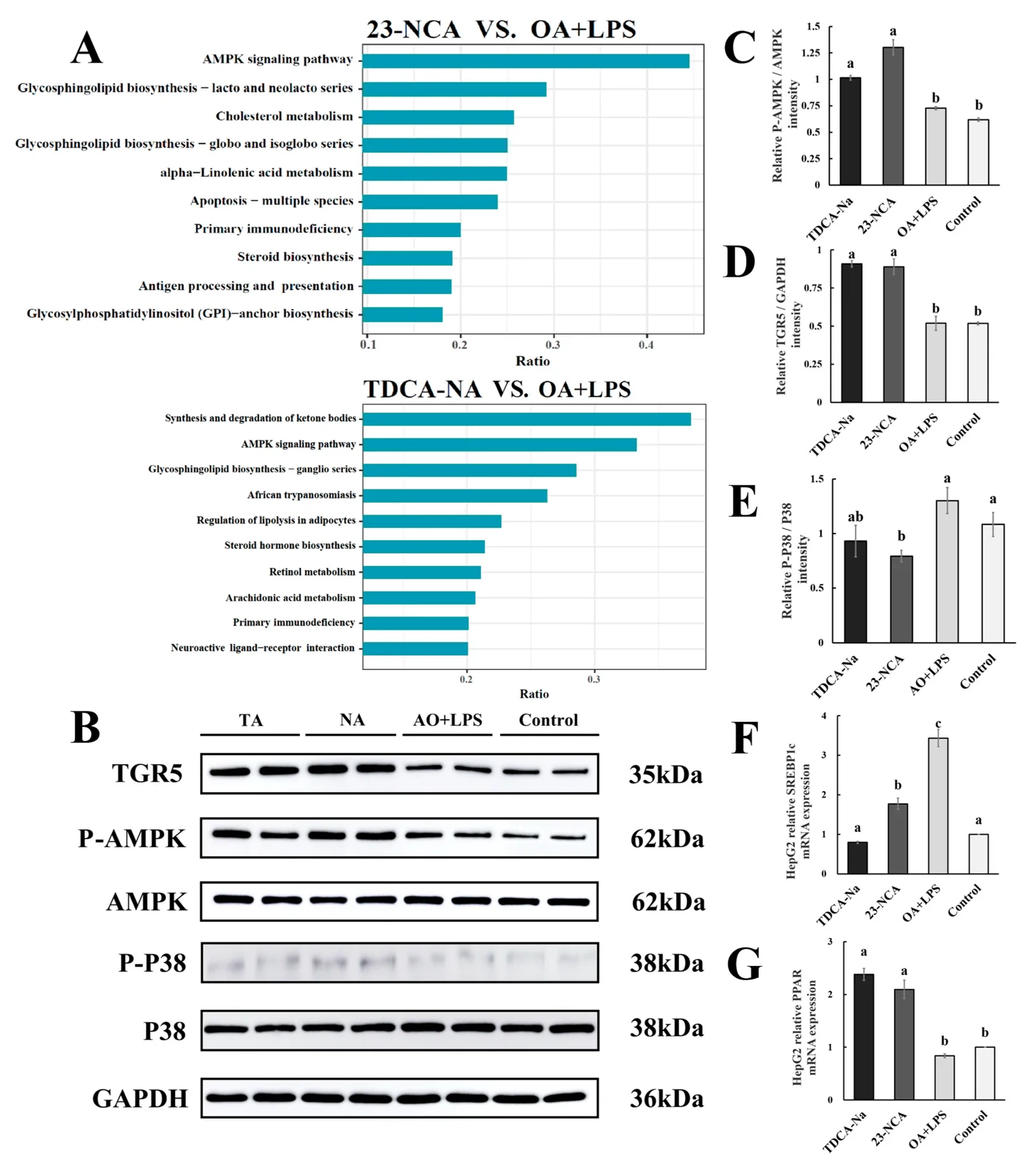
Identification of the core signaling pathway mediating the hepatoprotective effects of secondary bile acids. (**A**) KEGG pathway enrichment analysis of differentially expressed proteins identified by data-independent acquisition (DIA) quantitative proteomics in hepatocytes treated with 23-NCA versus the OA+LPS model group (top panel) and TDCA-Na versus the OA+LPS model group (bottom panel), with the horizontal axis representing the enrichment ratio (n = 3). (**B**) Western blot analysis of TGR5, phosphorylated AMPK (P-AMPK), total AMPK, phosphorylated P38 (P-P38), and total P38 protein expression; GAPDH was used as the loading control. (**C**–**E**) Quantitative analysis of relative protein expression levels: (**C**) P-AMPK/AMPK, (**D**) TGR5/GAPDH, and (**E**) P-P38/P38. (**F**,**G**) Relative mRNA expression levels of (**F**) SREBP-1c and (**G**) PPAR were measured by qRT-PCR. For Western blotting (**C**–**E**), four independent biological replicates were prepared per group and pooled pairwise before electrophoresis, yielding two pooled samples per group for densitometric analysis. For (**C**–**G**), different letters indicated significant differences between groups (*p* < 0.05) (n = 4).

To elucidate the molecular mechanisms underlying the hepatoprotective effects of 23-NCA and TDCA-Na, we performed data-independent acquisition (DIA) quantitative proteomic profiling to compare protein expression in control, OA+LPS model, and 23-NCA- or TDCA-Na-treated hepatocytes. KEGG pathway enrichment analysis of differentially expressed proteins revealed that the AMPK signaling pathway was significantly enriched in both 23-NCA vs. OA+LPS and TDCA-Na vs. OA+LPS comparisons (Figure 5A), suggesting that it is the core regulatory target through which these secondary bile acids exert their protective effects. Western blot validation confirmed that, compared with the OA+LPS model group, both 23-NCA and TDCA-Na treatment significantly upregulated TGR5 receptor protein expression (Figure 5B,D), enhanced AMPK phosphorylation (elevated P-AMPK/AMPK ratio; Figure 5B,C), and suppressed P38 MAPK phosphorylation (reduced P-P38/P38 ratio; Figure 5B,E). These findings indicate that 23-NCA and TDCA-Na treatments are accompanied by TGR5 upregulation and coordinated modulation of the AMPK and P38 MAPK pathways, which may contribute to their hepatoprotective actions.

## 4. Discussion

This study provides a multi-layered characterization of gut microbiota alterations in MASLD, spanning taxonomic composition, co-occurrence network architecture, functional metabolic pathway shifts, and mechanistic validation through in vitro experiments. Our findings provide an integrated framework linking MASLD-associated gut dysbiosis, centered on the depletion of *Bifidobacterium longum* as a keystone species, to the reduced predicted genomic potential for secondary bile acid biosynthesis and, ultimately, to the identification of two specific SBAs, 23-NCA and TDCA-Na, that protect hepatocytes concomitant with modulation of the TGR5–AMPK signaling pathway [43,44].

The cross-validation strategy using four complementary analytical methods (LEfSe, STAMP, ALDEx2, and XGBoost) addresses a common limitation in microbiome studies, where single-method differential abundance testing can yield false positives driven by data sparsity or compositionality effects [30,31]. The consistent identification of *B. longum* across all four methods (LEfSe, STAMP, ALDEx2, and XGBoost), combined with its top-ranked feature importance in the machine learning model (highest SHAP value = 0.628), provides robust evidence that this organism represents a primary discriminative feature of the MASLD-associated gut microbiota. The loss of *B. longum* in MASLD is consistent with previous reports of reduced *Bifidobacterium* abundance in metabolic liver disease [7,8,45]; however, our network analysis extends this observation by demonstrating that *B. longum* depletion is accompanied by a marked decline in its network centrality (degree centrality reduced from 34 to 20; betweenness centrality from 0.023 to 0.009) and a shift toward predominantly negative interactions. This pattern suggests that MASLD disrupts not merely the taxonomic composition but the ecological architecture of the gut microbiota, with the collapse of a keystone species potentially reflecting or contributing to the destabilization of the broader microbial community [28,46].

The functional significance of *B. longum* depletion is further supported by the positive correlation between its abundance and the SBA biosynthesis pathway, which was significantly downregulated in the MASLD gut microbiota. *Bifidobacterium* species, including *B. longum*, are known to express bile salt hydrolase (BSH) enzymes that catalyze the deconjugation of primary bile acids, a rate-limiting step in the generation of secondary bile acids by the gut microbiota [12,20,47]. The concurrent reduction in *B. longum* abundance and SBA pathway activity therefore suggests a mechanistic link: the loss of this keystone BSH-active species is likely associated with a compromised gut microbiota’s capacity to produce SBAs, potentially depriving the liver of bile acid species with signaling functions in metabolic regulation [11,12,48,49].

Our in vitro screening demonstrated that 23-NCA and TDCA-Na exhibited potential protective properties, significantly attenuating inflammatory cytokine release and partially reducing hepatocellular lipid accumulation, although the extent of steatosis reversal was modest compared to the suppression of inflammatory markers. The proteomic and Western blot evidence demonstrated TGR5 upregulation concomitant with enhanced AMPK phosphorylation and suppression of P38 MAPK signaling. This TGR5–AMPK–P38 axis is biologically plausible: TGR5 is a G protein-coupled receptor for secondary bile acids that has been implicated in energy homeostasis and inflammation [13,14,19], and AMPK is a central metabolic sensor whose activation improves hepatic steatosis and insulin sensitivity [16,17]. P38 MAPK, in contrast, is a stress-responsive kinase whose activation contributes to inflammation and apoptosis in hepatocytes [19]. The coordinated modulation of these two pathways, AMPK activation and P38 suppression, by 23-NCA and TDCA-Na may explain their ability to simultaneously address both the steatotic and inflammatory components of the MASLD phenotype [50].

Several limitations of this study should be acknowledged. First, the in vitro HepG2 model, while widely used for hepatocyte studies, may not fully recapitulate the metabolic complexity of primary human hepatocytes or the multicellular liver microenvironment [51]. Second, the PICRUSt2-predicted functional pathway alterations, although consistent with the observed taxonomic shifts, require direct confirmation through metatranscriptomic, metabolomic, or metaproteomic approaches [35,52]. Third, while the *B. longum*–*Clostridium* cross-feeding axis supports overall secondary bile acid biosynthetic potential, the specific bacterial taxa directly generating 23-NCA and TDCA-Na in vivo remain to be characterized through future metabolomic and gnotobiotic studies. Fourth, the causal relationship between *B. longum* depletion, altered circulating SBA profiles, and hepatocellular protection has not been directly validated in vivo; targeted metabolomic profiling and gnotobiotic animal experiments are required to establish causality beyond in vitro observations [28,46]. Fifth, despite standardized DADA2 preprocessing and four-way orthogonal validation (LEfSe, STAMP, ALDEx2, and XGBoost), random 5-fold cross-validation on merged cohorts carries residual overfitting risks, underscoring the need for external prospective cohorts to validate B. longum as a diagnostic biomarker.

Despite these limitations, our findings have several implications. The identification of *B. longum* as a putative keystone species whose network centrality collapses in MASLD together with shifts in other dominant taxa (such as Faecalibacterium) suggests that future therapeutic strategies might need to consider broader ecological network restoration [5,6,53]. Furthermore, the TGR5–AMPK–P38 signaling axis identified here provides a molecular framework for the development of SBA-based or SBA-analog therapeutics for MASLD [14,16]. Future studies should investigate whether 23-NCA and TDCA-Na exert similar protective effects in in vivo MASLD models, and whether the restoration of *B. longum* abundance, through prebiotic, probiotic, or dietary interventions, can rescue SBA biosynthesis and ameliorate MASLD progression [20,21,48].

## Figures and Tables

**Table 2:** Topological parameters of the gut microbiota co-occurrence networks in the healthy and MASLD groups.

Statistics	Health	MASLD
**Ecological Interactions**		
Average shortest path length	1.782	1.825
Network density	0.242	0.223
Prevalence of Negative Interactions	0.521541	0.518814
**Robustness**		
Clustering coefficient	0.440	0.439
Network heterogeneity	0.587	0.630
Network centralization	0.370	0.436
***Bifidobacterium longum***		
Degree	34	20
Clustering coefficient	0.241	0.221
Betweenness Centrality	0.023	0.009
Prevalence of Negative Interactions	0.462	0.531

MASLD, Metabolic dysfunction-associated steatotic liver disease.

## Data Availability

All data generated or analyzed in the current study are available in the figshare repository (https://doi.org/10.6084/m9.figshare.33045686 (accessed on 27 September 2026)). There are no restrictions on any datasets used, and other researchers can access all of them.

## Supplementary Materials

The following supporting information can be downloaded at: https://www.mdpi.com/article/10.3390/microorganisms14102315/s1. File S1: Attachment Ethics Review Form and WB.

## References

[1] Rinella M.E., Lazarus J.V., Ratziu V., Francque S.M., Sanyal A.J., Kanwal F., Romero D., Abdelmalek M.F., Anstee Q.M., Arab J.P. (2023). A multisociety Delphi consensus statement on new fatty liver disease nomenclature. Hepatology.

[2] Riazi K., Azhari H., Charette J.H., E Underwood F., A King J., Afshar E.E., Swain M.G., E Congly S., Kaplan G.G., Shaheen A.-A. (2022). The prevalence and incidence of NAFLD worldwide: A systematic review and meta-analysis. Lancet Gastroenterol. Hepatol..

[3] Younossi Z.M., Golabi P., Paik J.M., Henry A., Van Dongen C., Henry L. (2023). The global epidemiology of nonalcoholic fatty liver disease (NAFLD) and nonalcoholic steatohepatitis (NASH): A systematic review. Hepatology.

[4] Eslam M., Newsome P.N., Sarin S.K., Anstee Q.M., Targher G., Romero-Gomez M., Zelber-Sagi S., Wong V.W.-S., Dufour J.-F., Schattenberg J.M. (2020). A new definition for metabolic dysfunction-associated fatty liver disease: An international expert consensus statement. J. Hepatol..

[5] Fan Y., Pedersen O. (2021). Gut microbiota in human metabolic health and disease. Nat. Rev. Microbiol..

[6] Tilg H., Adolph T.E., Trauner M. (2022). Gut-liver axis: Pathophysiological concepts and clinical implications. Cell Metab..

[7] Sharpton S.R., Ajmera V., Loomba R. (2019). Emerging Role of the Gut Microbiome in Nonalcoholic Fatty Liver Disease: From Composition to Function. Clin. Gastroenterol. Hepatol..

[8] Aron-Wisnewsky J., Vigliotti C., Witjes J., Le P., Holleboom A.G., Verheij J., Nieuwdorp M., Clément K. (2020). Gut microbiota and human NAFLD: Disentangling microbial signatures from metabolic disorders. Nat. Rev. Gastroenterol. Hepatol..

[9] Mouries J., Brescia P., Silvestri A., Spadoni I., Sorribas M., Wiest R., Mileti E., Galbiati M., Invernizzi P., Adorini L. (2019). Microbiota-driven gut vascular barrier disruption is a prerequisite for non-alcoholic steatohepatitis development. J. Hepatol..

[10] Arrese M., Cabrera D., Kalergis A.M., Feldstein A.E. (2016). Innate immunity and inflammation in NAFLD/NASH. Dig. Dis. Sci..

[11] Jia W., Xie G., Jia W. (2018). Bile acid–microbiota crosstalk in gastrointestinal inflammation and carcinogenesis. Nat. Rev. Gastroenterol. Hepatol..

[12] Guzior D.V., Quinn R.A. (2021). Microbial transformations of human bile acids. Microbiome.

[13] Kawamata Y., Fujii R., Hosoya M., Harada M., Yoshida H., Miwa M., Fukusumi S., Habata Y., Itoh T., Shintani Y. (2003). A G protein-coupled receptor responsive to bile acids. J. Biol. Chem..

[14] Perino A., Velázquez-Villegas L.A., Bresciani N., Sun Y., Huang Q., Fénelon V.S., Castellanos-Jankiewicz A., Zizzari P., Bruschetta G., Jin S. (2021). Central anorexigenic actions of bile acids are mediated by TGR5. Nat. Metab..

[15] Agus A., Clément K., Sokol H. (2021). Gut microbiota-derived metabolites as central regulators in metabolic disorders. Gut.

[16] Steinberg G.R., Carling D. (2019). AMP-activated protein kinase: The current landscape for drug development. Nat. Rev. Drug Discov..

[17] Herzig S., Shaw R.J. (2018). AMPK: Guardian of metabolism and mitochondrial homeostasis. Nat. Rev. Mol. Cell Biol..

[18] Song X., Sun X., Oh S.F., Wu M., Zhang Y., Zheng W., Geva-Zatorsky N., Jupp R., Mathis D., Benoist C. (2020). Microbial bile acid metabolites modulate gut RORγ+ regulatory T cell homeostasis. Nature.

[19] Canovas B., Nebreda A.R. (2021). Diversity and versatility of p38 kinase signalling in health and disease. Nat. Rev. Mol. Cell Biol..

[20] Wong C.B., Odamaki T., Xiao J.-Z. (2019). Beneficial effects of Bifidobacterium longum subsp. longum BB536 on human health: Modulation of gut microbiome as the principal action. J. Funct. Foods.

[21] Rivière A., Selak M., Lantin D., Leroy F., De Vuyst L. (2016). Bifidobacteria and butyrate-producing colon bacteria: Importance and strategies for their stimulation in the human gut. Front. Microbiol..

[22] Page M.J., McKenzie J.E., Bossuyt P.M., Boutron I., Hoffmann T.C., Mulrow C.D., Shamseer L., Tetzlaff J.M., Akl E.A., Brennan S.E. (2021). The PRISMA 2020 statement: An updated guideline for reporting systematic reviews. BMJ.

[23] Bolyen E., Rideout J.R., Dillon M.R., Bokulich N.A., Abnet C.C., Al-Ghalith G.A., Alexander H., Alm E.J., Arumugam M., Asnicar F. (2019). Reproducible, interactive, scalable and extensible microbiome data science using QIIME 2. Nat. Biotechnol..

[24] Callahan B.J., Mcmurdie P.J., Rosen M.J., Han A.W., Johnson A.J.A., Holmes S.P. (2016). DADA2: High-resolution sample inference from Illumina amplicon data. Nat. Methods.

[25] Quast C., Pruesse E., Yilmaz P., Gerken J., Schweer T., Yarza P., Peplies J., Glöckner F.O. (2012). The SILVA ribosomal RNA gene database project: Improved data processing and web-based tools. Nucleic Acids Res..

[26] R Core Team (2021). R: A Language and Environment for Statistical Computing.

[27] Lê S., Josse J., Husson F. (2008). FactoMineR: An R package for multivariate analysis. J. Stat. Softw..

[28] Liu R., Hong J., Xu X., Feng Q., Zhang D., Gu Y., Shi J., Zhao S., Liu W., Wang X. (2017). Gut microbiome and serum metabolome alterations in obesity and after weight-loss intervention. Nat. Med..

[29] Wickham H. (2016). Data analysis. Ggplot2: Elegant Graphics for Data Analysis.

[30] Segata N., Izard J., Waldron L., Gevers D., Miropolsky L., Garrett W.S., Huttenhower C. (2011). Metagenomic biomarker discovery and explanation. Genome Biol..

[31] Chen T., Guestrin C. XGBoost: A scalable tree boosting system. Proceedings of the 22nd ACM SIGKDD International Conference on Knowledge Discovery and Data Mining.

[32] Lundberg S.M., Lee S.-I. (2017). A unified approach to interpreting model predictions. Adv. Neural Inf. Process. Syst..

[33] Watts S.C., Ritchie S.C., Inouye M., E Holt K. (2019). FastSpar: Rapid and scalable correlation estimation for compositional data. Bioinformatics.

[34] Bastian M., Heymann S., Jacomy M. Gephi: An open source software for exploring and manipulating networks. Proceedings of the International AAAI Conference on Web and Social Media.

[35] Douglas G.M., Maffei V.J., Zaneveld J.R., Yurgel S.N., Brown J.R., Taylor C.M., Huttenhower C., Langille M.G.I. (2020). PICRUSt2 for prediction of metagenome functions. Nat. Biotechnol..

[36] Kanehisa M., Furumichi M., Sato Y., Kawashima M., Ishiguro-Watanabe M. (2023). KEGG for taxonomy-based analysis of pathways and genomes. Nucleic Acids Res..

[37] Jiang G., Zhang J., Zhang Y., Yang X., Li T., Wang N., Chen X., Zhao F.-J., Wei Z., Xu Y. (2023). DCiPatho: Deep cross-fusion networks for genome scale identification of pathogens. Brief. Bioinform..

[38] Meroni M., Paolini E., Longo M., Piciotti R., Tria G., Fargion S., Fracanzani A.L., Dongiovanni P. (2022). Recreating gut-liver axis during NAFLD onset by using a Caco-2/HepG2 co-culture system. Metab. Target Organ Damage.

[39] Chen J., Liu Y., Luo H., Chen G., Zheng Z., Wang T., Hu X., Zhao Y., Tang J., Su C. (2022). Inflammation Induced by Lipopolysaccharide and Palmitic Acid Increases Cholesterol Accumulation via Enhancing Myeloid Differentiation Factor 88 Expression in HepG2 Cells. Pharmaceuticals.

[40] Liang H., Zhang L., Wang H., Tang J., Yang J., Wu C., Chen S. (2015). Inhibitory Effect of Gardenoside on Free Fatty Acid-Induced Steatosis in HepG2 Hepatocytes. Int. J. Mol. Sci..

[41] An Z., Chen Q., Bu C., Chen L., Shi D., Zhang B., Jin L., Chi L. (2025). Structural Domain Analysis of Heparin and Heparan Sulfate Combined With Label-Free Quantitative Proteomics to Elucidate Their Functional Diversity in Liver Cancer and APAP-Induced Liver Injury. Mol. Cell. Proteom..

[42] IBM Corp (2017). IBM SPSS Statistics for Windows.

[43] Wahlström A., Sayin S.I., Marschall H.-U., Bäckhed F. (2016). Intestinal Crosstalk between Bile Acids and Microbiota and Its Impact on Host Metabolism. Cell Metab..

[44] Paik J.M., Golabi P., Younossi Y., Mishra A., Younossi Z.M. (2020). Changes in the Global Burden of Chronic Liver Diseases From 2012 to 2017: The Growing Impact of NAFLD. Hepatology.

[45] Sanyal A.J., Van Natta M.L., Clark J., Neuschwander-Tetri B.A., Diehl A., Dasarathy S., Loomba R., Chalasani N., Kowdley K., Hameed B. (2021). Prospective Study of Outcomes in Adults with Nonalcoholic Fatty Liver Disease. N. Engl. J. Med..

[46] Kolodziejczyk A.A., Zheng D., Shibolet O., Elinav E. (2019). The role of the microbiome in NAFLD and NASH. EMBO Mol. Med..

[47] Lv S., Huang J., Luo Y., Wen Y., Chen B., Qiu H., Chen H., Yue T., He L., Feng B. (2024). Gut microbiota is involved in male reproductive function: A review. Front. Microbiol..

[48] Hidalgo-Cantabrana C., Delgado S., Ruiz L., Ruas-Madiedo P., Sánchez B., Margolles A. (2018). Bifidobacteria and their health-promoting effects. Bugs as Drugs: Therapeutic Microbes for the Prevention and Treatment of Disease.

[49] Chiang J.Y.L., Ferrell J.M. (2018). Bile Acid Metabolism in Liver Pathobiology. Gene Expr..

[50] Arab J.P., Karpen S.J., Dawson P.A., Arrese M., Trauner M. (2017). Bile acids and nonalcoholic fatty liver disease: Molecular insights and therapeutic perspectives. Hepatology.

[51] Loomba R., Friedman S.L., Shulman G.I. (2021). Mechanisms and disease consequences of nonalcoholic fatty liver disease. Cell.

[52] Moszak M., Szulińska M., Walczak-Gałęzewska M., Bogdański P. (2021). Nutritional Approach Targeting Gut Microbiota in NAFLD—To Date. Int. J. Environ. Res. Public Heal..

[53] Schoeler M., Caesar R. (2019). Dietary lipids, gut microbiota and lipid metabolism. Rev. Endocr. Metab. Disord..

